# Attending to history in climate change–demography research

**DOI:** 10.1553/p-hzdz-jega

**Published:** 2024-09-09

**Authors:** Emily Klancher Merchant, Kathryn Grace

**Affiliations:** 1 UC Davis, Davis, California, USA; 2 University of Minnesota, Minneapolis, Minnesota, USA

**Keywords:** Climate change, Population, Demography, History, Environment

## Abstract

Climate change is among the most urgent challenges of our time. While often considered a problem for the natural and physical sciences, the humanities and social sciences have made equally important interventions into research on the reciprocal relationship between humans and our climate. Because demography occupies the intersection of the natural and social sciences, and because it deals specifically with rates of change in social and natural processes, we believe it can make valuable contributions to the pressing imperatives of understanding and addressing climate change and mitigating the harms it is already visiting on the world’s most vulnerable people. We also believe that climate change may afford demographers an opportunity to expand our capacity to think about time and space at finer scales, and to examine the relationships among the core demographic processes – mortality, fertility and migration – which have typically been considered in isolation from one another. Yet responsibly leveraging climate change to advance demography, and leveraging demography to advance climate science and policy, require a cognizance of history that will assist demographers and those who use our analyses in avoiding the replication of past harms and, we hope, the invention of new ones. Understanding the history of demography and of population-environment thought more broadly can help us challenge assumptions that have not served science or policy well in the past – such as the assumption that larger or faster-growing populations necessarily put more pressure on the environment, independent of structural conditions – and consider alternative theoretical framings that might lead to better scientific models and policy solutions.

## Introduction

It is impossible in some parts of the world to consider population dynamics without considering the impacts of climate change. Demographers, with their ability to analyze complex population data to consider the experience of an individual in context, bring important data, analytical skills and insight into discussions of how climate change and population interact. Examining the way human systems, in particular population, respond to different types of climate change events provides us with an opportunity to advance our understanding of behavioral and structural responses across scales, from the individual level up through some relevant aggregate level (e.g., community, livelihood zone or country).

Among the key tools demography has brought to bear on the intersection of climate and population are systems thinking and multi-level analysis (e.g., Mackinnon, 1995; Greenhalgh, 1990; Mason and Taj, 1987; Watkins, 1993; and many, many others). These approaches suggest that individuals make decisions within cultural or socioeconomic systems situated within spatial and temporal contexts, and that as these systems shift and change, the options for individuals also change. In turn, the contexts shift as individuals adopt and normalize new ways of doing things. In recent decades, demographers have utilized multi-level statistical models to identify effects operating at various levels, from the individual to the household or village to the ecological level (e.g., Fussell et al., 2014). However, the complexities of these analytic frameworks and methods have not really penetrated the interdisciplinary domain of climate change research. Indeed, it can even be argued that describing and quantitatively modeling the heterogeneity in multi-level human systems – i.e., the relationships between individuals, families, communities and beyond – and the dynamic feedback between population and the environment are vital, but understudied, components of climate change research with important implications for policy and science, which fail to account for the interplay between population and environment when they focus on only one level at a time or neglect recursive effects between levels (Peng et al., 2021; Hallegatte and Rozenberg, 2017; Grace, 2017).

While demographers are equipped with tools to analyze complex social systems and individual processes, the complexities of contemporary environmental and climate data pose data and methodological challenges. Climate change takes complex forms along a spatial and temporal spectrum, while demography, especially foundational population-environment demography, often relies on aggregate spatial units (e.g., country or urban/rural) and coarse temporal units (e.g., yearly, five-year, 10-year or even coarser units). For example, weekly shifts in vegetation during key crop growing seasons can have impacts on fertility behavior in the short term, as women are more likely to space their births differently in some settings (Brooks et al., 2023, Grace et al., 2021). Aggregating rainfall to seasonal or annual indicators would miss this relationship, and, because growing seasons vary within countries, aggregating rainfall data to a country-level average growing season would also muddy this relationship (Dorelien and Grace, 2023). Climate change happens both more slowly and more quickly than can be accounted for with an annual scale of analysis. For example, heat waves are as short as two or three days, while long-term warming occurs over decades or longer. In terms of geographic scales, communities interact with the landscape differently depending on a range of social and environmental factors that vary from community to community, but political responses to climate change can occur at a regional level (e.g., North America or the European Union), thus making country-level analyses only one of many useful scales. Rapid onset events – such as floods, landslides and wildfires – can erode agricultural productivity, cause destruction of shelter and pose infectious disease risks not only in the communities where these events occurred, but also in communities that are more geographically distal but have social or economic connections to the disaster sites. Slow onset events – such as gradual warming and drying, shortening of the growing season, rising sea levels and increasing rainfall intensity – may reduce the viability of livelihoods across regions, requiring significant investments in economic development at a multinational scale.

Regardless of the pace or geographic scale of threats, climate change impacts people’s daily lives – for example, by changing their subsistence strategies and childbearing plans – and has significant and lasting effects on individual behaviors, decision-making and health and well-being outcomes (e.g., Watts et al., 2018). These individual effects are likely to have emergent consequences at aggregate levels, with potential impacts on demographic composition and economic development (Fankhauser and Stern, 2016; Kaczan and Orgill-Meyer, 2020; Lutz, 2017), which will further impact individuals in a recursive cycle.

## Why history matters

As demographers and policy makers pay increasing attention to climate change as a driver of population change, it is imperative that we avoid replicating the past harms of demography and of environmental Malthusianism. In contrast to demography, which may be defined as the social science of human population dynamics, environmental Malthusianism refers to efforts to limit population growth in order to protect the natural environment (Merchant, 2022). Historically, environmental Malthusianism has been more popular among natural scientists than among demographers, many of whom have been suspicious of claims that population growth has any necessary, direct or straightforward effect on the natural environment (e.g., Coale, 1968; Mair, 1949; Notestein, 1970). By attending to the history of both demography and environmental Malthusianism, however, today’s demographers can leverage the experience of the past to improve our science and use it to advance effective policy today.

Environmental Malthusianism drew on the tradition of Thomas Robert Malthus, who wrote about population in England and worldwide at the turn of the 19^th^ century. Malthus had theorized that all human ills stemmed from the pressure of population on subsistence resources, and that only the delayed marriage customary of the English middle class could prevent poverty, famine and war. His *Essay on the Principle of Population* influenced the passage of England’s New Poor Law in 1834, which forced the poor into workhouses to curtail their childbearing (Wrigley and Smith, 2020).

Environmental Malthusianism emerged in the 1920s in the United States, when natural scientists pointed to soil erosion as evidence that the United States was overpopulated to support their calls for immigration restriction and a eugenics program aimed at reducing births among the poor (East, 1923; Pearl, 1922). It gained increasing popularity after World War II through the work of the eugenicist Guy Irving Burch (Burch and Pendell, 1947), the conservationist Fairfield Osborn (1948) and the ornithologist William Vogt (1948). Osborn and Vogt arguably launched the modern environmental movement, and focused it on the purported dangers of human population growth (Desrochers and Hoffbauer, 2009). In 1968, the biologist Paul Ehrlich brought environmental Malthusianism into the atomic age, arguing in his bestselling *Population Bomb* that rapid population growth would inevitably lead to nuclear war (Ehrlich, 1968). While Ehrlich himself ultimately disavowed eugenics and racism, his work and that of his allies, including the biologist Garret Hardin (1968), supported the development of eco-fascism, an ideology that uses environmentalism as an excuse for renewed immigration restriction (Bhatia, 2004; Normandin and Valles, 2015). The social critic Naomi Klein has termed it “environmentalism through genocide” (quoted in Corcione, 2020).

The problem with environmental Malthusianism is not that it can *lead to* eco-fascism, but rather that it is *rooted in* eugenics and racism (Merchant, 2022). Although many of the founding works of environmental Malthusianism (Burch and Pendell, 1947; East, 1923; Ehrlich, 1968; Hardin, 1968; Osborn, 1948; Pearl, 1922; Vogt, 1948) were published by scientists, they were not grounded in empirical evidence of a direct relationship between population growth and environmental harm. They demonstrated that specific activities – such as intensive agriculture, pesticide use, deforestation and suburban sprawl – had detrimental effects on the environment, and recommended reducing population as an alternative to regulating those activities. These works thus served as scientistic rationales for political agendas that were popular among certain groups of scientists: eugenics and immigration restriction in the first half of the 20^th^ century and population control in the Global South in the second half. This is not to say that climate change and other environmental catastrophes do not have human causes – they undoubtedly do. These disasters, however, are caused primarily by decisions made in legislative bodies and corporate boardrooms, with little direct relationship to the number of people on the planet (Sayre, 2012). Eco-fascism is therefore not an extreme reaction to scientific evidence that population growth is bad for the environment. There is currently no scientific consensus on the number of people the Earth can support, with estimates ranging from orders of magnitude fewer than we have now to orders of magnitude more (Pengra, 2012). Rather, eugenics and racism were the starting points for scientific claims that are backed by little empirical evidence, and that, at best, serve to divert political will away from support for environmental regulation and toward support for family planning. To be sure, family planning is an important component of health care. It is not, however, an adequate form of environmental protection.

Demographers have long been critical of and concerned about the overly simplified and facile links between population growth and ecosystem degradation posited by environmental Malthusians. For example, the 1949 meeting of the Population Association of America (PAA) devoted a dinner session to explicit critiques of Osborn and Vogt (Mair, 1949); the demographer Ansley Coale implicitly critiqued Ehrlich’s *Population Bomb* in his 1968 PAA presidential address (Coale, 1968); and the 1970 PAA meeting included a critical discussion of the environmental Malthusian organization Zero Population Growth, or ZPG for short (Notestein, 1970). A small number of demographers – most notably Kingsley Davis and some of his students – did get on the environmental Malthusian bandwagon (see, for example, Davis, 1948; Osborn and Davis, 1955a, b). Davis even joined the board of ZPG. Other demographers were not unconcerned about the environment, but recognized, as Coale put it in 1968, that even “a population one-half or three-quarters the size of the current one in the United States could ruin the potability of our fresh water supplies and poison our atmosphere by the unrestricted discharge of waste,” thereby challenging the idea that more people necessarily means more pollution (Coale, 1968, p. 470). These demographers preferred more direct forms of environmental regulation, such as cap-and-trade systems for pollutants.

Although demographers openly critiqued environmental Malthusianism among themselves in the late 1960s and early 1970s, they rarely made their opposition public because the widespread belief that population growth was bad for the environment generated popular support for demographers’ own population control agenda (Merchant, 2021). On the basis of a single simulation study conducted in the 1950s (Coale and Hoover, 1958), demographers convinced heads of state throughout the Global South that achieving economic development required reductions in fertility rates. Working from the unproven premise that rapid population growth posed a barrier to economic development, many demographers shifted their research agendas to the problem of how to get women in agrarian societies to adopt new contraceptive technologies (Merchant, 2017), thus contributing to complex narratives around poor people, and poor women in particular, that characterized them as simultaneously very powerful and very naïve (Sasser, 2018; Grace, 2017; Hodgson and Watkins, 1997; Watkins, 1993). In the 1960s, demographers also served as advisors to family planning programs with the explicit goal of reducing national birth rates, rather than meeting the contraceptive needs of individuals and couples (Takeshita, 2012). Although the excesses of these family planning programs are today often remembered as extreme responses to a real problem (Connelly, 2008), the history of demography indicates that the perceived problem of rapid population growth (as a barrier to economic development or a threat to the natural environment) was in fact constructed on the basis of incomplete data supplemented by racist assumptions (Greenhalgh, 1996; Hodgson, 1988; Szreter, 1993).

In the decades after World War II, the environmental Malthusianism of natural scientists and the economic Malthusianism of demography were both funded by U.S.-based corporate interests and driven by fears among American businessmen and diplomats that rapid population growth abroad – particularly in what were then known as the Second (communist) and Third (nonaligned) Worlds – threatened U.S. national security and global economic hegemony (Merchant, 2021). Intellectuals and heads of state from the Global South rejected environmental Malthusianism at the 1972 UN Conference on the Human Environment (Selcer, 2018) and rejected economic Malthusianism at the 1974 UN World Population Conference (Finkle and Crane, 1975). Demographic research in the 1980s demonstrated that there was no necessary or automatic relationship between population growth and economic growth or environmental degradation, as the relationship was always mediated by social, political and economic institutions (National Research Council, 1986). Nonetheless, many economists continued to push for fertility interventions as a means of promoting economic growth, calling for fertility reductions in the Global South to generate a “demographic dividend” (Foley, 2022), and, more recently, for an increase in fertility in the Global North to prevent the age structure from becoming too top-heavy (Spears, 2023). Environmental scientists, for their part, have continued to attribute ecosystem degradation, and now climate change, to growing human numbers (Royal Society of London and U.S. National Academy of Sciences, 1992; for a critique, see Sasser, 2018), and even some former critics of population control have begun to join them (Clarke and Haraway, 2018).

## Demography and the natural environment today

Demographers dabbled in questions of population and environment, mostly in indirect ways, during the 1980s, as studies of famine and demography emerged in response to the Sahelian famines of the late 1970s and concerns about the failures of humanitarian aid to successfully intervene. Famine was observed to be an outcome of drought, but also of failed economic and political systems (Watkins and Menken, 1985; Caldwell et al., 1986; Bongaarts, 1980; Faulkingham and Thorbahn, 1975). In general, demographers determined that famine as a preventative check on population growth did not occur, contrary to Malthusian ideas, and instead settled on the perspective that famines that were at least partially driven by environmental disasters had very little impact on births, but had some impact on deaths and migration. Proposed solutions to famine primarily focused on humanitarian and policy interventions that promoted food allocation and proper land management. Additional research focusing on the role of agriculture and labor, especially child labor (demand for children) and sometimes women’s labor (energetic output and fecundity), existed on the margins of the field (e.g., Panter-Brick, 1996; Lee and Kramer, 2002). For the most part, however, questions of how the (seasonally and spatially varying) natural environment impacts demographic processes were largely unexplored.

A notable shift occurred during the early 1990s and 2000s with the rise of a kind of conservation-oriented or sustainability-oriented demography that focused on the community and the micro levels (e.g., Bilsborrow, 1987; Bilsborrow and DeLargy, 1990; Pebley, 1998). Some of this research echoed earlier Malthusian ideas about resource scarcity, exploring the ways that migration and sometimes fertility contributed to environmental degradation. Around the same time, demographers converted the journal *Population and Environment* from an outlet for eco-fascist pseudoscience into a reputable demography journal (Merchant, 2022) that frequently published articles exploring the impact of population change (mostly growth) on land use. Macro-level research focusing on population growth and high fertility, especially as a problem for global environmental health and climate change, also rapidly expanded (e.g., O’Neill et al., 2001; O’Neill et al., 2010).

Exploiting spatial detail in health surveys, questions about the micro-level effects of a dynamic natural environment on population processes, especially with a focus on women’s and children’s lives, began to slowly emerge in the 2010s (Grace et al., 2012; Grace, 2017; Isen et al., 2017). The changing data infrastructure, in combination with a growing interest in exploring how individual-level biology and behavior changed in relation to local weather and environmental conditions, helped to inspire the expansion of geographic approaches to analysis in demography (in terms of both quantitative and qualitative methods), and motivated efforts to combine human and ecological data in new ways and at multiple scales (e.g., Grace et al., 2021; Bakhtsiyarava et al., 2018; Dorelien and Grace, 2023; Brooks et al., 2023; Randell et al. 2021).

Current research indicates that the demographic effects of climate change are pervasive, and can be difficult to isolate from other demographic processes. For example, heat-related mortality tends to be higher in communities with older age structures, which already have higher mortality levels. Similarly, climate change can exacerbate preterm labor, especially in communities that already have higher levels of infant mortality. Repeated miscarriages or stillbirths associated with heat stress may also shift childbearing goals and perceptions of risk around infant health. As with other external events (e.g., conflict or infectious disease), how an individual experiences and responds to climate change depends on the severity, duration and frequency of the climate event; the demographic composition of the community; and the individual’s role in the community at that time (e.g., Grace, 2017; Hill et al., 2019). Research shows that climate change can exacerbate social inequalities – from micro to macro levels – because impacts are experienced differently both between and within communities (Rao et al., 2019; Lau et al., 2021; Brooks et al., 2023; Hallegatte and Rozenberg, 2017). Additionally, how humans biologically, behaviorally and systematically (through infrastructure and policy) respond to climate change now has implications for future generations (Hondula et al., 2015). For example, children may benefit if their care providers have learned to strategically manage resources to ensure household health as a result of having experienced repeated droughts (Hill et al., 2019; Isen et al., 2017). Alternatively, community- or county-level food shipping or storage practices may exacerbate risks for certain communities and individuals, limiting the suite of available household coping strategies and resulting in adverse health impacts for children (Grace et al., 2016). For all of these reasons, it is imperative for demographers to further develop their analytic capacity to examine how climate, climate events and climate change influence patterns of fertility, mortality, health and migration, while considering all of these processes in dynamic relationship to the natural world, as more and more demographers are beginning to do. As we do so, however, it is crucial that we remember the history of our field and of the larger population movement so that we can learn from past mistakes and avoid replicating past harms or creating novel harms.

Going forward, it is vital to build a climate change-demography approach that integrates the critiques from the past with the needs of the future to ensure that demographic science helps to improve the lives of people now and the lives of future generations. The history of demography and environmental Malthusianism indicates that fertility should not be understood as a means to an end. Reducing fertility will not, in and of itself, protect the environment or create a demographic dividend. Nor will increasing fertility, in and of itself, generate economic growth. Social, political and economic institutions always mediate between population, economies and environments, and it is to these institutions – not to family planning or pronatalism – that we should turn to produce the economic or environmental outcomes we desire. History suggests that doing demography responsibly requires that the field commits to the tenets of reproductive justice, a theoretical and political framework developed by women of color – who have historically been the targets of coercive pro- and anti-natalist programs – that promotes the ability of *individuals* to have the children they want to have; to not have the children they do not want to have; and to raise the children they do have under safe and dignified circumstances (Ross and Solinger, 2017). Research on the relationship between population and climate change has the potential to meaningfully contribute to the third of these aims. By understanding how climate change influences demographic processes, we can help policy makers anticipate and provide for the needs of the world’s most vulnerable families. This only becomes possible, however, when we stop blaming families themselves and the survival strategies they undertake in a changing climate for causing climate change, and instead address the structural causes of our current crisis, from global capitalism to environmental racism.

## References

[R1] Bakhtsiyarava M, Grace K, and Nawrotzki RJ (2018). Climate, birth weight, and agricultural livelihoods in Kenya and Mali. American Journal of Public Health, 108(S2), S144–S150. 10.2105/AJPH.2017.304128 29072943 PMC5922197

[R2] Bhatia R (2004). Green or brown? White nativist environmental movements. In Ferber AL (Ed.), Home-grown hate (pp. 208–228). Routledge.

[R3] Bilsborrow RE (1987). Population pressures and agricultural development in developing countries: A conceptual framework and recent evidence. World Development, 15(2), 183–203. 10.1016/0305-750X(87)90077-5

[R4] Bilsborrow RE, and DeLargy PF (1990). Land use, migration, and natural resource deterioration: The experience of Guatemala and the Sudan. Population and Development Review, 16 (Supplement: Resources, Environment, and Population: Present Knowledge, Future Options), 125–147. 10.2307/2808067

[R5] Bongaarts J (1980). Does malnutrition affect fecundity? A summary of evidence. Science, 208 (4444), 564–569. 10.1126/science.7367878 7367878

[R6] Brooks N, Grace K, Kristiansen D, Shukla S, and Brown ME (2023). Investigating the relationship between growing season quality and childbearing goals. Global Environmental Change, 80, 102677. 10.1016/j.gloenvcha.2023.102677 37250477 PMC10209476

[R7] Burch GI, and Pendell E (1947). Human breeding and survival. Penguin Books. 10.1097/00010694-194804000-00011

[R8] Caldwell JC, Reddy PH, and Caldwell P (1986). Periodic high risk as a cause of fertility decline in a changing rural environment: Survival strategies in the 1980–1983 South Indian drought. Economic Development and Cultural Change, 34(4), 677–701. 10.1086/451554

[R9] Clarke AE, and Haraway D (Eds.) (2018). Making kin, not population. Prickly Paradigm Press.

[R10] Coale AJ (1968). Should the United States start a campaign for fewer births? Population Index, 34(4), 467–474. 10.2307/2732649

[R11] Coale AJ, and Hoover EM (1958). Population growth and economic development in low-income countries: A case study of India’s prospects. Princeton University Press.

[R12] Connelly M (2008). Fatal misconception: The struggle to control world population. Harvard University Press.

[R13] Corcione A (2020). Eco-fascism: What it is, why it’s wrong, and how to fight it. Teen Vogue, 30 April 2020. https://teenvogue.com/story/what-is-ecofascism-explainer

[R14] Davis K (1948). Population and resources in the Americas. In Proceedings of the Inter-American Conference on Conservation of Renewable Natural Resources (pp. 88–97). U.S. Department of State.

[R15] Desrochers P, and Hoffbauer C (2009). The post-war intellectual roots of the population bomb: Fairfield Osborn’s ‘Our Plundered Planet’ and William Vogt’s ‘Road to Survival’ in retrospect. The Electronic Journal of Sustainable Development, 1(3), 73–97.

[R16] Dorelien A, and Grace K (2023). Climate change-related demographic and health research: Data and approaches. In Ortiz SE, McHale SM, King V, and Glick JE (Eds.), Environmental impacts on families (pp. 43–63). Springer. 10.1007/978-3-031-22649-6_3

[R17] East EM (1923). Mankind at the crossroads. Charles Scribner’s Sons.

[R18] Ehrlich P (1968). The population bomb. Ballantine Books.

[R19] Fankhauser S, and Stern N (2016). Climate change, development, poverty and economics. In Basu K, Rosenblatt D, Sepúlveda S (Eds.). The state of economics, the state of the world. MIT Press. 10.7551/mitpress/11130.003.0012

[R20] Faulkingham RH, and Thorbahn PF (1975). Population dynamics and drought: A village in Niger. Population Studies, 29(3), 463–477. 10.1080/00324728.1975.10412711 22077779

[R21] Finkle JL, and Crane BB (1975). The politics of Bucharest: Population, development, and the New International Economic Order. Population and Development Review, 1(1), 87–114. 10.2307/1972272

[R22] Foley E (2022). In pursuit of the demographic dividend: The return of economic justifications for family planning in Africa. Sexual and Reproductive Health Matters, 30(1), 216–230. 10.1080/26410397.2022.2133352 PMC9621287 36305801

[R23] Fussell E, Hunter LM, and Gray CL (2014). Measuring the environmental dimensions of human migration: The demographer’s toolkit. Global Environmental Change, 28, 182–191. 10.1016/j.gloenvcha.2014.07.001 25177108 PMC4144443

[R24] Grace K (2017). Considering climate in studies of fertility and reproductive health in poor countries. Nature Climate Change, 7(7), 479–485. 10.1038/nclimate3318 PMC6009846 29937922

[R25] Grace K, Davenport F, Funk C, and Lerner AM (2012). Child malnutrition and climate in Sub-Saharan Africa: An analysis of recent trends in Kenya. Applied Geography, 35(1–2), 405–413. 10.1016/j.apgeog.2012.06.017

[R26] Grace K, Nagle NN, and Husak G (2016). Can small-scale agricultural production improve children’s health? Examining stunting vulnerability among very young children in Mali, West Africa. Annals of the American Association of Geographers, 106(3), 722–737. 10.1080/24694452.2015.1123602

[R27] Grace K, Verdin A, Dorélien A, Davenport F, Funk C, and Husak G (2021). Exploring strategies for investigating the mechanisms linking climate and individual-level child health outcomes: An analysis of birth weight in Mali. Demography, 58(2), 499–526. 10.1215/00703370-8977484 33834220 PMC8382135

[R28] Greenhalgh S (1990). Toward a political economy of fertility: Anthropological contributions. Population and Development Review, 16(1), 85–106. 10.2307/1972530

[R29] Greenhalgh S (1996). The social construction of population science: An intellectual, institutional, and political history of twentieth-century demography. Comparative Studies in Society and History, 39(1), 26–66. 10.1017/S0010417500020119

[R30] Hallegatte S, and Rozenberg J (2017). Climate change through a poverty lens. Nature Climate Change, 7(4), 250–256. 10.1038/nclimate3253

[R31] Hardin G (1968). The tragedy of the commons. Science, 162(3859), 1243–1248. 10.1126/science.162.3859.1243 5699198

[R32] Hill R, Skoufias E, and Maher B (2019). The chronology of a disaster: A review and assessment of the value of acting early on household welfare. World Bank. 10.1596/31721

[R33] Hodgson D (1988). Orthodoxy and revisionism in American demography. Population and Development Review, 14(4), 541–569. 10.2307/1973624

[R34] Hodgson D, and Watkins SC (1997). Feminists and neo-Malthusians: Past and present alliances. Population and Development Review, 23(3), 469–523. 10.2307/2137570

[R35] Hondula DM, Balling RC, Vanos JK, and Georgescu M (2015). Rising temperatures, human health, and the role of adaptation. Current Climate Change Reports, 1(3), 144–154. 10.1007/s40641-015-0016-4

[R36] Isen A, Rossin-Slater M, and Walker R (2017). Relationship between season of birth, temperature exposure, and later life wellbeing. Proceedings of the National Academy of Sciences, 114(51), 13447–13452. 10.1073/pnas.1702436114 PMC5754756 29203654

[R37] Kaczan DJ, and Orgill-Meyer J (2020). The impact of climate change on migration: a synthesis of recent empirical insights. Climatic Change, 158(3), 281–300. 10.1007/s10584-019-02560-0

[R38] Lau JD, Kleiber D, Lawless S, and Cohen PJ (2021). Gender equality in climate policy and practice hindered by assumptions. Nature Climate Change, 11(3), 186–92. 10.1038/s41558-021-00999-7

[R39] Lee RD, and Kramer KL (2002). Children’s economic roles in the Maya family life cycle: Cain, Caldwell, and Chayanov revisited. Population and Development Review, 28(3), 475–499. 10.1111/j.1728-4457.2002.00475.x

[R40] Lutz W (2017). How population growth relates to climate change. Proceedings of the National Academy of Sciences, 114(46), 12103–12105. 10.1073/pnas.1717178114 PMC5699101 29093163

[R41] Mackinnon A (1995). Were women present at the demographic transition? Questions from a feminist historian to historical demographers. Gender & History, 7(2), 222–240. 10.1111/j.1468-0424.1995.tb00022.x 12346528

[R42] Mair GF (Ed.) (1949). Studies in population: Proceedings of the annual meeting of the Population Association of America at Princeton, NJ, May 1949. Princeton University Press.

[R43] Mason KO, and Taj AM (1987). Differences between women’s and men’s reproductive goals in developing countries. Population and Development Review, 13(4), 611–638. 10.2307/1973025

[R44] Merchant EK (2022). Environmental Malthusianism and demography. Social Studies of Science, 52(4), 536–560. 10.1177/03063127221104929 35735176 PMC9315181

[R45] Merchant EK (2021). Building the population bomb. Oxford University Press. 10.1093/oso/9780197558942.001.0001

[R46] Merchant EK (2017). A digital history of Anglophone demography and global population control, 1915–1984. Population and Development Review, 43(1), 83–117. 10.1111/padr.12044

[R47] National Research Council. (1986). Population growth and economic development: Policy questions. National Academies Press. 10.17226/620

[R48] Normandin S, and Valles SA (2015). How a network of conservationists and population control activists created the contemporary U.S. anti-immigration movement. Endeavour, 39(2), 95–105. 10.1016/j.endeavour.2015.05.001 26026333

[R49] Notestein FW (1970). Zero Population Growth. Population Index, 36(4), 444–452. 10.2307/2734065 12277271

[R50] O’Neill BC, MacKellar FL, and Lutz W (2001). Population and climate change. Cambridge University Press. 10.1017/CBO9780511529450

[R51] O’Neill BC, Dalton M, Fuchs R, Jiang L, Pachauri S, and Zigova K (2010). Global demographic trends and future carbon emissions. Proceedings of the National Academy of Sciences, 107(41), 17521–17526. 10.1073/pnas.1004581107 PMC2955139 20937861

[R52] Osborn HF Jr. (1948). Our plundered planet. Little, Brown and Company.

[R53] Osborn HF Jr., and Davis K (1955a). Food and people: U.S. farm surpluses are no answer to the world’s food shortages The Wall Street Journal, 7 April 1955.

[R54] Osborn HF Jr., and Davis K (1955b). Space and people: Migration to U.S. cannot relieve world’s overcrowding. The Wall Street Journal, 8 April 1955.

[R55] Panter-Brick C (1996). Proximate determinants of birth seasonality and conception failure in Nepal. Population Studies, 50(2), 203–220. 10.1080/0032472031000149306 11613333

[R56] Pearl R (1922). World overcrowding: Saturation point for Earth’s population soon will be in sight, with the safety limit for the United States estimated at 200,000,000 people—How the nations grow men much like flies. High cost of unfit. The New York Times, 8 October 1922. https://nytimes.com/1922/10/08/archives/world-overcrowding-saturation-point-for-earths-population-soon-will.html

[R57] Pebley AR (1998). Demography and the environment. Demography, 35(4), 377–389. 10.2307/3004008 9850464

[R58] Peng W, Iyer G, Bosetti V, Chaturvedi V, Edmonds J, Fawcett AA, Hallegatte S, Victor DG, van Vuuren D, and Weyant J (2021). Climate policy models need to get real about people-here’s how: To predict how society and political systems might actually respond to warming, upgrade integrated assessment models. Nature, 594(7862), 174–176. 10.1038/d41586-021-01500-2 34103720 PMC9531597

[R59] Pengra B (2012). One planet, how many people? A review of Earth’s carrying capacity. UNEP Global Environmental Alert Service (GEAS). June 2012. https://na.unep.net/geas/archive/pdfs/GEAS_Jun_12_Carrying_Capacity.pdf

[R60] Randell H, Grace K, and Bakhtsiyarava M (2021). Climatic conditions and infant care: Implications for child nutrition in rural Ethiopia. Population and Environment, 42(4), 524–552. 10.1007/s11111-020-00373-3 34149138 PMC8210853

[R61] Rao N, Lawson ET, Raditloaneng WN, Solomon D, and Angula MN (2019). Gendered vulnerabilities to climate change: Insights from the semi-arid regions of Africa and Asia. Climate and Development, 11(1), 14–26. 10.1080/17565529.2017.1372266

[R62] Ross L, and Solinger R (2017). Reproductive justice: An introduction. University of California Press.

[R63] Royal Society of London and U.S. National Academy of Sciences. (1992). Population growth, resource consumption, and a sustainable world. London and Washington: Royal Society of London and U.S. National Academy of Sciences.

[R64] Sasser J (2018). On infertile ground: Population control and women’s rights in the era of climate change. New York University Press.

[R65] Sayre NF (2012). The politics of the anthropogenic. Annual Review of Anthropology, 41, 57–70. 10.1146/annurev-anthro-092611-145846

[R66] Selcer P (2018). The postwar origins of the global environment: How the United Nations built spaceship Earth. Columbia University Press. 10.7312/selc16648

[R67] Spears D (2023). The world’s population may peak in your lifetime. What happens next? The New York Times, 18 September 2023. https://nytimes.com/interactive/2023/09/18/opinion/human-population-global-growth.html

[R68] Szreter S (1993). The idea of demographic transition and the study of fertility change: A critical intellectual history. Population and Development Review, 19(4), 659–701. 10.2307/2938410

[R69] Takeshita C (2012). The global biopolitics of the IUD: How science constructs contraceptive users and women’s bodies. MIT Press.

[R70] Vogt W (1948). Road to survival. Victor Gollancz, Ltd.

[R71] Watkins SC (1993). If all we knew about women was what we read in *Demography*, what would we know? Demography, 30(4), 551–577. 10.2307/2061806 8262280

[R72] Watkins SC, and Menken J (1985). Famines in historical perspective. Population and Development Review,11(4), 647–675. 10.2307/1973458

[R73] Watts N, Amann M, Ayeb-Karlsson S, Belesova K, Bouley T, Boykoff M, Byass P, Cai W, Campbell-Lendrum D, Chambers J, Cox PM, Daly M, Dasandi N, Davies M, Depledge M, Depoux A, Dominguez-Salas P, Drummond P, Elkins P … and Costello A (2018). The Lancet countdown on health and climate change: From 25 years of inaction to a global transformation for public health. The Lancet, 391(10120), 581–630. 10.1016/S0140-6736(17)32464-9 29096948

[R74] Wrigley EA, and Smith R (2020). Malthus and the poor law. The Historical Journal, 63(1), 33–62. 10.1017/S0018246X19000177

